# School Diversity Perspectives and Classmate Diversity Climate: Understanding High School Students' School Belonging, Well‐Being, and Intercultural Contact

**DOI:** 10.1002/jcop.70082

**Published:** 2026-01-02

**Authors:** Jana Vietze, Nina Stuur, Joep Hofhuis

**Affiliations:** ^1^ Erasmus University Rotterdam Rotterdam the Netherlands; ^2^ Amsterdam University of Applied Sciences Amsterdam the Netherlands

**Keywords:** adolescence, diversity climate, ethnic minority, intercultural contact, school belonging, school diversity perspectives, well‐being

## Abstract

This study examined how school diversity perspectives (multiculturalism, critical consciousness, color‐evasion) may enhance student outcomes (school belonging, well‐being, intercultural contact) and whether a strong diversity climate among classmates (openness and appreciation of diversity) may moderate this link. We surveyed *N* = 280 high school students (*M*_age_ = 15.95; 47.5% female; 1.8% nonbinary) in the Netherlands. We performed multigroup structural equation modeling, comparing cultural minority (*n* = 81) and majority (*n* = 203) students, and multilevel analysis to separate individual‐level from classroom‐level effects. A strong diversity climate among classmates and color‐evasion in school (not multiculturalism and critical consciousness) were robustly, positively related to belonging and well‐being (minority and majority students) and intercultural contact (minority students). For all students, a strong diversity climate among classmates amplified the relation between color‐evasion with student outcomes. Findings highlight the need to distinguish school‐level policies from classroom‐level practices in addressing educational inequities between minority and majority students.

## 1 Introduction

The Netherlands is an increasingly ethnically and culturally diverse society, due to an influx of migrants from different parts of the globe and their descendants. The resulting ethnic and cultural diversity among the Dutch population has a significant impact on the educational system, which is slow to respond with necessary educational policies (Solano and Huddleston 2020). In schools, both the benefits and challenges of diversity can be felt on a daily basis, and schools are challenged to address educational inequities in the classroom while providing students with the necessary skills to participate in an increasingly diverse society (Inspectie van het Onderwijs 2022). Recent work in the field of educational science has identified *cultural diversity climate* in schools as important driver of positive student outcomes, including intergroup attitudes and relationships, academic outcomes, and socioemotional outcomes (for a review, see Bardach et al. 2024). However, this concept has been conceptualized in different ways, making it impossible to formulate concrete implications for how schools can ultimately approach structural and educational inequities (Celeste et al. 2019). On school level, diversity climate has captured more general approaches for addressing cultural, ethnic, and or racial diversity (e.g., Byrd 2017; Celeste et al. 2019; Thapa et al. 2013). On classroom level, it has captured a variety of facets including diversity‐related teaching content and how students from different cultural groups interact with each other (e.g., Schachner et al. 2021). While each approach has led to meaningful insights regarding students' optimal educational experiences and development, we believe that previous studies may have overestimated the extent to which school‐level approaches are mirrored in classroom‐level practices.

The present paper aims to examine if school‐level and classroom‐level concepts may have divergent or enhancing effects on cultural minority and majority student outcomes (school belonging, general well‐being, intercultural contact), and whether they may even interact to predict student outcomes. To do so, we are drawing from previous literature in organizational science^1^, where a clear distinction is made between *cultural diversity perspectives*, meaning higher‐level ideologies on how to best manage diversity (Ely and Thomas 2001; Podsiadlowski et al. 2013), and *diversity climate*, which reflects how groups of same‐level individuals (e.g., classmates) are interacting and how diversity is enacted among these individuals (Hofhuis et al. 2012; Mckay et al. 2007).

### 1.1 School Belonging, Well‐Being, and Intercultural Contact Among Cultural Minority and Majority Students

Based on previous literature, the present study employs three distinct student outcomes that together provide an overview of the degree to which a school is successful in dealing with cultural diversity in the classroom. These outcomes represent the extent to which students show desirable socioemotional outcomes (i.e., “feeling well”) and intergroup outcomes (i.e., “doing well,” Suárez‐Orozco et al. 2018). First, we investigate *school belonging*, which is defined as the extent to which students feel personally accepted, included, liked, respected, and supported by others in their school environment (Goodenow 1993). Previous research shows that a sense of school belonging is an important predictor of academic emotional and behavioral engagement (Gillen‐O'Neel 2021), as well as overall well‐being of students (Jose et al. 2012). Whereas a meta‐analysis by Allen et al. (2018) found no mean differences across 51 studies between minority and majority students, school belonging may have a different, more central function for the development of educational outcomes (e.g., efficacy and ambitions) for students from minority compared to majority ethnic and cultural groups (Murphy and Zirkel 2015). A possible reason is that minority students are more likely to feel disrespected, less attached, or more alienated from school due to perceived societal pressures and negative stereotypes regarding their own ethnic or cultural group (Steele 1997).

Second, we investigate general, subjective *well‐being* which is a person's global assessment of their own life quality according to their own criteria (Diener et al. 1985) and an important predictor of future mental health (Keyes et al. 2010). For both minority and majority students, the school context can contribute significantly to well‐being, next to school belonging, for example, by fostering connections among students (Polk et al. 2020). Especially for cultural minority youth, identifying with school can predict positive developments in well‐being (Bratt 2015). Therefore, subjective well‐being is an important indicator of how well students feel in general, but also in their educational environment.

Third, we investigate *intercultural contact*, generally defined as the contact and friendships among students of different ethnic, religious, or cultural backgrounds (e.g., Schachner et al. 2021), focusing on the quality of interactions and collaborations that take place between students in the classroom environment. Under the right conditions, which can be fostered by schools, intercultural contact with peers is seen as a strong predictor of positive diversity attitudes (Allport 1954; Pettigrew and Tropp 2008). On the flipside, schools can be the place for experiencing negative intergroup contact, such as higher perceived discriminatory treatment by school personnel and peers, which is related to ethnic minority students' poorer academic performance and psychological adjustment (Benner and Graham 2013).

Below, we will outline how school cultural diversity perspectives and diversity climate among classmates may be linked with school belonging, well‐being, and intercultural contact among minority and majority students.

### 1.2 School Cultural Diversity Perspectives and Positive Student Outcomes

Outside the field of education, diversity perspectives have been extensively studied in the context of organizations, which has led to a rich body of work on their effects on employee interactions and well‐being (for a review, see Hofhuis and Vietze 2025). Similar to organizations, schools can be considered organizational units that formulate diversity policies—based on higher‐level ideologies on how to best manage diversity (i.e., diversity perspectives, Ely and Thomas 2001; Podsiadlowski et al. 2013)—with the goal to manage and address diversity among teaching staff and students (Celeste et al. 2019). Therefore, it is not surprising that there is a conceptual overlap between unique—but not mutually exclusive—cultural diversity perspectives that both organizations and schools commonly adopt in their goal to manage diversity. The present study focuses on three specific diversity perspectives that have previously received attention in both organizational as well as educational research.

The first is *multiculturalism*, which is the belief that culture, race, and ethnicity should be recognized as meaningful and that group differences should be valued, for example, in culture‐sensitive learning materials in school valuing. Importantly, multiculturalism explicitly allows for, or even encourages, cultural minority individuals to identify with their own in‐groups in their social environment, for example, when students are affirmed in or learn about their own culture in school (Bardach et al. 2024). On organizational level, research has shown that multiculturalism indeed leads to more positive and secure identities (Verkuyten 2006), enhances inclusion, sense of belonging, and performance of ethnic and cultural minority individuals (Derks et al. 2007; Jansen et al. 2016), and allows all individuals to express their diverse experiences and viewpoints (Zhan 2023). In classrooms that promote multiculturalism, students are likely to report high intercultural contact (Juang et al. 2020). On school level, multiculturalism is likely related to positive socioemotional and academic outcomes of all students (Bardach et al. 2024) and to smaller belonging and achievement gaps between minority and majority students over time (Celeste et al. 2019).

A second common perspective is *critical consciousness*, which is the belief that social inequity should be actively discussed and reflected upon to promote awareness of and self‐efficacy to change injustices. This perspective has been identified as a successful way to address cultural differences in secondary schools (Heberle et al. 2020; Schwarzenthal et al. 2022) and in higher education (e.g., Cadenas et al. 2022; Sommier et al. 2022). It embodies the philosophy that awareness of unequal power distribution is essential for empowering minoritized individuals, and for valorizing individual differences (e.g., Zanoni et al. 2010). On classroom level, this perspective likely promotes desirable intergroup outcomes (e.g., intended critical action) for minority and majority students (Schwarzenthal et al. 2022), whereas on a school level, these effects may not be as detectable (Bardach et al. 2024).

A third common diversity perspective is *color‐evasion/colorblindness*, defined as the belief that de‐emphasizing ethnic or racial group memberships may reduce prejudice and discrimination (Cho et al. 2018; Civitillo et al. 2021). Advocates of color‐evasive ideology claim neutrality or objectiveness toward differences, focusing on similarity and cohesion, therefore *stressing similarity* (Civitillo et al. 2021). However, other scholars have argued that this ideology has another side to it, namely colorblindess, when it constitutes a denial of the complexity of a diverse social environment (Markus et al. 2000; Sommier et al. 2019) and is, therefore, *ignoring difference* (Civitillo et al. 2021). In fact, colorblindness has been related to increased intergroup bias (Richeson and Nussbaum 2004) and scholars have criticized the argument that the color‐evasive perspective can be neutral or objective (e.g., Cho et al. 2018), because it does not consider historic nor structural inequities.

In the school context, recent studies in Europe have replicated this conceptual difference between colorblindness and color‐evasion in schools: whereas a colorblind perspective in school may be related to lower school achievement among minority students (e.g., Phalet and Baysu 2020) and wider belonging and achievement gaps between minority and majority students over time (Celeste et al. 2019), there may be a positive association between school‐ and classroom‐level color‐evasion with a wide array of high school student outcomes, such as academic engagement, intercultural contact, intercultural competence, and global identity resolution (e.g., Juang et al. 2020; Schwarzenthal et al. 2020). A possible explanation for the latter could be that in Western Europe students and teachers may be more inclined to *stress similarities*, stemming from a positive, inclusion‐oriented intention to treat all students equally within a classroom (Civitillo et al. 2021). However, more empirical evidence is needed to support this assumption across European contexts.

### 1.3 A Strong Diversity Climate Among Classmates and Cultural Minority Status as Moderators

In addition to examining the influence of school‐level diversity perspectives, as explained above, the present study also focuses on how students are interacting with peers with different cultural or ethnic heritages in the classroom, termed *diversity climate among classmates*. Our definition of the concept *diversity climate* is based on previous research in the workplace context, where openness to and appreciation of diversity represent the two components of a strong diversity climate (see Hofhuis et al. 2012; 2016). First, *openness to diversity* is the possibility of students to freely discuss their heritages and display culturally relevant behaviors. Second, *appreciation of diversity* encompasses the perception that differences in students' heritages provide added value to the classroom environment and are seen as positive. Although these components can be measured separately, earlier studies which use this definition show that they correlate strongly and should be viewed as two aspects of one overarching construct of diversity climate (see also Dwertmann et al. 2016). This type of diversity climate, consisting of openness and appreciation of diversity in a social environment, has repeatedly been shown to relate to favorable outcomes such as quality of interactions, inclusion, and creativity in groups (Boehm et al. 2014; Gonzalez and Denisi 2009; Hofhuis et al. 2016).

Whereas recent studies from Europe have used diversity climate as umbrella term to comprise diversity perspectives *and* the way these perspectives are reflected in educational practices from different agents, including teachers and classmates (Bardach et al. 2024; Schachner et al. 2021), we believe that in schools, the distinction between perspectives and practices might be meaningful. A long tradition of studies in organizations (for a review, see Hofhuis and Vietze 2025) gives us reason to believe that there is a conceptual, and necessary distinction between diversity perspectives, which reflect a school′s ideology (i.e., how school personnel think diversity *should ideally be dealt with*), and diversity climate, which reflects how diversity is perceived and lived by students in the classroom on a day‐to‐day basis (i.e., how students among themselves *are dealing* with diversity). In fact, ample literature on diversity climate in organizations supports that an organization's diversity perspective is fairly independent from team diversity climate, as is typically indicated by large variation of perceived diversity climates (compared to less variation in perceived diversity perspectives) within teams of the same organization (e.g., Dwertmann et al. 2016; Gonzalez and Denisi 2009; Hofhuis et al. 2012). Also, educational literature has shown that perceived school diversity perspectives are often incongruent between different agents within the same school, such as teachers and students (Civitillo et al. 2017; Fine‐Davis and Faas 2014; Konings et al. 2024), which may be the result of aggregating constructs at different levels. Therefore, instead of assuming that classroom‐level practices are a necessary representation of school‐level perspectives, we separately measure both constructs in the current study.

**Figure 1:**
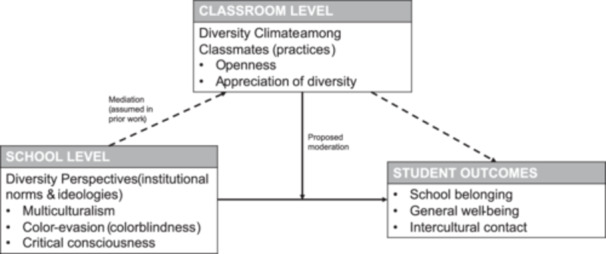
Conceptual model illustrating the proposed relationships between school‐level diversity perspectives, diversity climate between classmates, and student outcomes.

Moreover, we test if a strong diversity climate among classmates may be a necessary condition for perceived school diversity perspectives (multiculturalism, critical consciousness, color‐evasion) to have a positive effect on student outcomes. Based on the assumed conceptual independence of diversity climate and diversity perspectives, we believe that any school diversity perspective can only be meaningfully related to positive student outcomes, if classmates are open to and appreciative of cultural diversity (i.e., a strong diversity climate among classmates). In case of a low openness and appreciation of cultural diversity (i.e., a weak diversity climate among classmates), we expect that neither school‐level perspective is related to student outcomes. In other words, the school‐level perspective may not matter if classmates are unsupportive of diversity to begin with. In this study, we therefore test if a strong diversity climate among classmates may moderate, and thus amplify the positive relationship between school diversity perspectives and student outcomes (see Figure 1).

In the field of organizational psychology, there is some research that shows that being part of a cultural or ethnic minority may moderate the effect of multiculturalism and color‐evasion on job outcomes (Jansen et al. 2016) and of diversity climate on job outcomes (Hofhuis et al. 2012). However, in the school context, a recent meta‐analysis found no significant differences between minority and majority students when relating diversity perspectives to overall student outcomes, combining socioemotional, academic, and intergroup outcomes (Bardach et al. 2024). To provide additional evidence, we explore whether the expected links between school diversity perspectives and diversity climate among classmates with student outcomes differ between cultural minority and cultural majority students.

### 1.4 The Current Study

In the present study, we investigate the relation between school diversity perspectives (multiculturalism, critical consciousness, color‐evasion) and cultural minority and majority students' socioemotional outcomes (school belonging, general well‐being) and intergroup outcomes (intercultural contact). All three diversity perspectives have been positively related—to varying extent—to minority and majority students' socioemotional development, but not intergroup outcomes in Western Europe (for a meta‐analysis, see Bardach et al. 2024). In this study, we first test whether these assumptions hold in high schools in the Netherlands, a country which has *halfway favorable* educational policies toward migrants and their descendants (Solano and Huddleston 2020) and has politically shifted from multiculturalism to assimilation and—due to recent rises in populism—to restriction of migration altogether (Jennissen et al. 2023; Scholten 2025). Next, we test whether a strong diversity climate among classmates and cultural minority status may moderate the relation between school diversity perspectives and student outcomes. We formulated the following hypotheses:

School cultural diversity perspectives (multiculturalism, critical consciousness, color‐evasion) are positively related to students' socioemotional development (school belonging, general well‐being) and unrelated to the intergroup outcome (intercultural contact among classmates).

These effects are amplified if students perceive a positive—compared to a negative—diversity climate (openness and appreciation of diversity) among classmates.

Direct and moderating effects are similar for cultural minority and majority students.

## 2 Materials and Methods

### 2.1 Procedure

The abovementioned hypotheses were tested using a quantitative survey study among students enrolled in secondary education in the Netherlands. Since respondents were under the age of 18, therefore informed consent was obtained from both parents and school management before data collection and from the respondents themselves during data collection. All procedures were approved by the Ethics Review Board of the authors' institution, prior to the sampling and recruitment procedure.

The survey was distributed under the supervision of the second author on paper during regular school hours, with the teacher being present. The second author gave a short introduction about the research topic and its procedures before the start of data collection. It was emphasized that answers would be anonymous and would not be shared with teachers or parents. In classrooms where roughly half of the students or more had obtained parental approval, the survey was conducted with all the students present. The students whose parents had not responded or had not given approval were present in the classroom as well and were asked to do a different task in silence so as not to disturb the participating students. In classrooms where less than half of the students were allowed to participate, these students were taken to a different room to complete the survey there. After the survey was conducted, the responses were manually digitalized.

### 2.2 Sampling Strategy

Because the survey contained school‐ and classroom‐level items next to individual‐level items, the aim was to recruit students from the same classroom and to include multiple classrooms from the same school. Therefore, the students were recruited through their teachers, who facilitated the participation of multiple of their classes. We specifically targeted teachers who were teaching courses related to social studies/civics (*maatschappijleer*), history, or geography in the last years of high school, assuming that these teachers and their students would be more familiar with the concepts of this study and better able to reflect on issues surrounding cultural diversity (Schwarzenthal et al. 2017). The teachers were recruited through snowball sampling. Three of the eight teachers who cooperated in this study came from the second and third author's personal networks, but the researcher collecting the data (Author 2) did not know any of the participants personally.

### 2.3 Sample

The final sample included *N* = 280 high school students (*M*_age_ = 15.95; 47.5% female, 1,8% nonbinary) located in urban areas in the West of the Netherlands. Students were sampled in 20 classrooms, across 6 high schools, with on average 13.4 students participating per classroom. The final sample included *n* = 81 cultural minority students, meaning that at least one parent or students themselves had been born outside of the Netherlands, and *n* = 203 cultural majority students, meaning that they and their parents had been born in the Netherlands. Cultural minority students reported family heritages from 38 different countries, representing large ethnic and cultural minority groups in the Netherlands, such as the Turkish (4.2% of the full sample), Surinamese (4.2%), and Moroccan minority group (3.5%). 60.6% attended the theory‐oriented *vwo* track, 37.7% attended the intermediate *havo* track, and 1.8% attended the practice‐oriented *vmbo* track.

Out of the initial sample (*N* = 284 students), 4 students had been removed from the data set prior to analysis because we had < 5 student responses from their classrooms and our analyses relied heavily on the nested classroom structure.

### 2.4 Measures

Surveys were administered in Dutch. To measure the constructs below, either prevalidated Dutch scales were used, or scales were translated to Dutch by the researchers if Dutch scales were unavailable. The survey items were proof‐read and checked for comprehensibility by a secondary school teacher, to ensure that the items would be understandable for all participants. Except for some demographic questions, students responded on a 7‐point Likert scale ranging from *totally disagree* (1) to *totally agree* (7). A full list of items can be found via OSF (link: https://osf.io/576yc/).

#### 2.4.1 School Diversity Perspectives

This 18‐item measure was based on selected subscales of the Classroom Cultural Diversity Climate Scale (Schachner et al. 2021). Where necessary, items were reformulated to fit the Dutch context and to focus only on the school‐level instead of classroom‐level perspectives. Our scale included three subscales: First, *multiculturalism* encompassed perceived school visions and practices around valuing cultural and ethnic group differences that exist within the student population, and around teaching students about their own cultural and ethnic backgrounds (based on the original subscale: “heritage and intercultural learning”). It was measured with seven items (e.g., “In school we learn about the heritage cultures of fellow students”). *Critical consciousness* entailed perceived school visions and practices around the discussion of and reflection upon social inequities in society (original subscale: “critical consciousness”) and was measured with five items (e.g., “In school we talk about how people from different backgrounds do not always have the same opportunities in the Netherlands”). *Color‐evasion* measured perceived school visions and practices about de‐emphasizing cultural or ethnic group memberships and differences (based on the original scale: “color‐evasion”) and included six items (e.g., “In school we are taught that cultural differences do not play a role for who we are”). Exploring the factor structure, CFA revealed that a 3‐factor solution was acceptable, meaning that all three subscales loaded on individual factors. We also performed CFA (1‐factor solution) individually for each subscale multiculturalism, critical consciousness, and color‐evasion. Item factor loadings in all scales were between 0.63 and 0.86. Therefore, we included all items in the analyses. The reliabilities were good for multiculturalism (*α* = 0.80), critical consciousness (*α* = 0.86), and color‐evasion (*α* = 0.88).

#### 2.4.2 Diversity Climate Among Classmates

This six‐item measure was based on the Dutch scale used by Hofhuis et al. (2012), adapted from the organizational to the classroom context. It consisted of three items measuring openness toward diversity (e.g., “In my classroom, cultural differences between students are openly discussed”) and three items measuring appreciation of diversity (e.g., “In this classroom, we value differences in cultural background”), as perceived by the individual student. A low mean score indicated a weak diversity climate, whereas a high score indicated a strong diversity climate in the classroom. The reliability was adequate (*α* = 0.79).

#### 2.4.3 School Belonging

This nine‐item scale measured the extent to which students perceived a personal sense of being included, liked, and respected in their school. Items were selected from the original Class Belonging and Support Scale (Goodenow 1993) and reformulated to address the school instead of classroom context. Example items were “Other students in this school are very friendly to me” and “I feel like I am really part of my school.” The reliability was adequate (*α* = 0.77).

#### 2.4.4 Well‐Being

This five‐item scale measured subjective global life satisfaction using a Dutch translation of the Satisfaction With Life Scale (Diener et al. 1985). Example items were “I am happy with my life” and “Everything considered, my life is almost ideal.” The reliability was good (*α* = 0.87).

#### 2.4.5 Intercultural Contact Between Classmates

This 6‐item scale measured the individually perceived quality of interactions among classmates from different cultural heritages, and was a combination of the subscales “contact and cooperation” and “(un)equal treatment” of the Classroom Cultural Diversity Climate Scale (Schachner et al. 2021). Example items were “In my class, students from different cultural backgrounds get along well with one another” and “Some students in our class talk badly about students from other cultural backgrounds.” The reliability was adequate (*α* = 0.74).

#### 2.4.6 Cultural Minority Status

We created this dummy variable based on students' self‐reported country of birth of themselves and up to two parents, following the most recent definition of the Dutch Central Bureau of Statistics (Central Bureau for Statistics 2022). Students with at least one parent (or themselves) born outside of the Netherlands were coded as *cultural minority* (i.e., family heritage outside of the Netherlands; 1). If students themselves and all indicated parents were born in the Netherlands, they were coded as *cultural majority* (i.e., Dutch family heritage; 0).

#### 2.4.7 Control Variables

We assessed participants' self‐reported gender (“I am a…”), with the answer options *boy* (1), *girl* (2), *nonbinary or other* (3), and *I′d rather not say* (4). We also assessed participants' age in years (“What age are you?”).

### 2.5 Positionality Statement

All authors engage in research aimed at education equity and improving intercultural education for adolescents (Authors 1 and 3) and emerging adults (Authors 1 and 2). The authors occupied multiple shared identities and social positions, including cisgender, Western European, feminist, and highly educated (university/doctoral degree). We partly shared social identities and experiences with the students; for example, of having been part of a cultural minority in primary school (Author 1) and high school (Authors 1 and 2). Yet, we also recognize that our team lacked diversity and representation in other areas; for example, no author identified as Person of Color, second‐ or third‐generation migrant.

### 2.6 Analysis Plan

For our preliminary analyses, we first investigated correlations between study variables and control variables in SPSS. Second, we established measurement invariance of all study variables for cultural minority versus cultural majority students (H3), using MPlus (version 8.9; Muthén and Muthén 1998). Measurement invariance between cultural minority and cultural majority students showed that it was possible to establish scalar invariance for diversity perspectives (color‐evasion, multiculturalism, critical consciousness) and for all outcome variables (school belonging, well‐being, and intercultural contact), and partial scalar invariance for diversity climate. These findings allowed us to compare both means and relations between predictor and outcome variables between both groups, minority and majority students.

As part of our main analyses, we tested three separate multigroup structural equation models (SEM) using MPlus (version 8.9; Muthén and Muthén 1998), one for each outcome variable (school belonging, well‐being, intercultural contact). In these models, next to direct paths between diversity perspectives and outcomes (H1), we included diversity climate as moderator (H2) and compared minority and majority students (H3) while accounting for the nested structure of students in classrooms (TYPE = COMPLEX command). To identify differences between minority and majority students, we tested which paths of the SEM could be constrained (i.e., defined as similar) across groups, while maintaining an acceptable model fit. We assessed model fit using the comparative fit index (CFI) and the root mean squared error of approximation (RMSEA), with CFIs > 0.95 and RMSEAs < 0.06 indicating a good model fit (Hu and Bentler 1999). Due to limited degrees of freedom, the final models did not include control variables.

Finally, as sensitivity analyses, we explored whether it was warranted to disentangle within‐person from between‐person effects. We calculated intraclass correlation coefficients (ICCs), meaning the proportion of total variance per variable that is between classrooms (i.e., between‐person) compared to within students (i.e., within‐person). ICCs were high for intercultural contact (0.29) but relatively low for well‐being (0.02) and school belonging (0.05). Therefore, we performed additional multilevel analyses with intercultural contact as outcome variable and with diversity perspectives, diversity climate, and minority status as predictors on Level 1, and aggregated (on classroom‐level) perceptions of diversity perspectives and diversity climate on Level 2. To address H1, the model also included within‐level interactions between diversity climate and each of the three school diversity perspectives on Level 1. To address H3, the model also included within‐level interactions between cultural minority status and diversity climate and between cultural minority status and each of the three school diversity perspectives on Level 1. Again, due to limited degrees of freedom, the model did neither include control variables beyond minority status, nor three‐way interactions between school diversity perspectives, diversity climate, and minority status.

## 3 Results

### 3.1 Preliminary Analyses

Table 1 depicts the descriptives and correlations among all study variables, including control variables, separately for cultural minority and majority students. Compared to minority students, majority students reported on average higher school diversity perspectives (multiculturalism, critical consciousness, color‐evasion) and higher outcome variables (school belonging, well‐being, intercultural contact). For all students, school diversity perspectives were moderately positively correlated with one another (*r* = 0.35–0.61, *p* < 0.01). For majority, not minority students, all perspectives were also weakly to moderately positively correlated with diversity climate among classmates (*r* = 0.26–0.42, *p* < 0.01). For all students, school belonging was moderately positively correlated with well‐being (*r* = 0.58, *p* < 0.01, for minority students; *r* = 0.49, *p* < 0.01, for majority students) and with intercultural contact (*r* = 0.46, *p* < 0.01, for minority students; *r* = 0.33, *p* < 0.01, for majority students).

**Table 1:** Descriptive statistics and bivariate correlations among study variables.

	1	2	3	4	5	6	7	8	9
1 Age	—	0.06	−0.07	−0.31**	−0.09	−0.09	−0.29*	−0.15	0.12
2 Girls (vs. boys)	−0.06	—	0.14	0.12	0.00	−0.01	−0.13	−0.20	−0.12
3 Multiculturalism^a^	−0.14*	0.08	—	0.45**	0.61**	0.20	0.19	0.02	−0.05
4 Critical consciousness^a^	−0.22**	0.13	0.56**	—	0.38**	0.09	0.15	0.08	−0.07
5 Color‐evasion^a^	−0.18*	0.14	0.39**	0.35**	—	0.00	0.34**	0.20	0.13
6 Diversity climate among classmates	−0.11	0.15*	0.36**	0.26**	0.42**	—	0.34**	0.24*	0.21
7 School belonging	−0.08	0.06	0.07	0.18*	0.20**	0.21**	—	0.58**	0.46**
8 Well‐being	−0.07	−0.09	0.00	0.10	0.07	0.05	0.49**	—	0.20
9 Intercultural contact	−0.08	0.09	0.17*	0.08	0.37**	0.49**	0.33**	0.15*	—
*M* (SD) minority students	16.21 (0.86)	1.62 (0.52)	3.75 (1.02)	3.52 (1.20)	4.21 (1.20)	4.70 (0.98)	4.99 (0.93)	4.89 (1.22)	5.34 (1.07)
*M* (SD) majority students	15.84 (0.76)	1.50 (0.54)	3.94 (1.05)	3.73 (1.37)	4.50 (1.23)	4.65 (1.10)	5.30 (0.85)	5.14 (1.20)	5.44 (0.93)

*Note:* Correlations amongst cultural minority students (*n* = 81) above and correlations amongst majority students (*n* = 279) below the diagonal.

^a^ School diversity perspectives.

* *p* < 0.05;

** *p* < 0.01.

### 3.2 Multigroup Structural Equation Modeling for School Belonging, Well‐Being, and Intercultural Contact

The full results and coefficients of the three final multigroup SEMs are depicted in Table 2. For minority students, a higher proportion of the variance in school belonging could be explained by the predictors, compared to majority students. The opposite was the case regarding the explained variance of intercultural contact, which was higher for the majority of students. For well‐being, there was no difference in explained variance (for more details, see Table 2).

**Table 2:** Results of the three multigroup regression models (school belonging, well‐being, intercultural contact).

	School belonging			Well‐being			Intercultural contact		
*R* ^2^	0.26***/0.11**			0.04**/0.06**			0.12***/0.28***		
	*b*	SE	*β*	*b*	SE	*β*	*b*	SE	*β*
Multiculturalism (MC)^a^	−0.13	0.07	−0.13/−0.15	−0.18	0.12	−0.15/−0.15	−0.08	0.07	−0.07/−0.08
Critical consciousness (CC)^a^	0.10	0.07	0.09/0.12	0.15	0.09	0.12/0.13	−0.08	0.09	−0.06/−0.08
Color‐evasion (CB)^a^	**0.39** *** **/0.15** *	**0.10/0.06**	**0.40** *** **/0.17** *	0.16*	0.06	0.13*/0.13*	0.24**	0.08	0.22**/0.26**
Diversity climate among classmates (DC)	**0.38** *** **/0.14** *	**0.07/0.06**	**0.36** *** **/0.17** **	0.1	0.09	0.07/0.08	0.38***	0.07	0.33***/0.42***
MC × DC	−0.03	0.05	−0.03/−0.04	−0.06	0.1	−0.04/−0.06	0.06	0.07	0.04/0.07
CB × DC	0.15***	0.04	0.14**/0.21**	0.18**	0.07	0.13**/0.17*	0.08	0.08	0.06/0.09
CC × DC	−0.06	0.05	−0.05/−0.07	−0.10	0.09	−0.07/−0.09	−0.07	0.06	−0.05/−0.08

*Note:* Coefficients of minority students on the left, majority students on the right of the dash. Parameters freed to vary between minority and majority students in bold.

^a^ School diversity perspectives.

* *p* < 0.05;

** *p* < 0.01;

*** *p* < 0.001.

**Figure 2:**
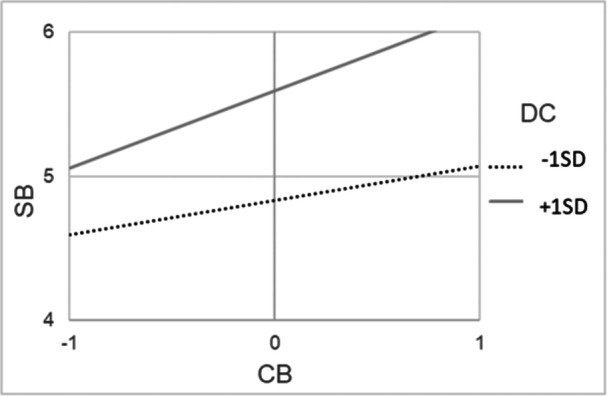
Interaction effects among cultural minority students between perceived school‐level color‐evasion (CB) and diversity climate among classmates (DC) in the multigroup structural equation model with school belonging (SB) as outcome.

**Figure 3:**
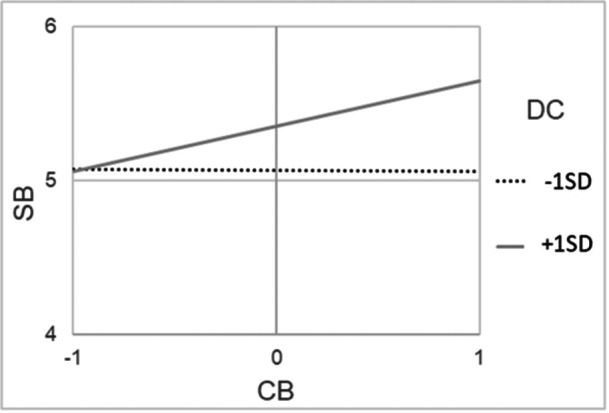
Interaction effects among cultural majority students between perceived school‐level color‐evasion (CB) and diversity climate among classmates (DC) in the multigroup structural equation model with school belonging (SB) as outcome.

Regarding school belonging, the model with two parameters freed (color‐evasion→school belonging; diversity climate→school belonging) was accepted as the most restrictive model with a good fit (*χ* ^2^/df = 1.05, *p* = 0.39, RMSEA = 0.02 [90% CI from 0.00 to 0.11], CFI = 0.99). Only one school diversity perspectives, namely color‐evasion, was significantly positively related to students' school belonging, and more strongly for minority students (*β* = 0.40, *p* < 0.001) compared to majority students (*β* = 0.17, *p* = 0.02). Also, diversity climate was positively, directly related to students' school belonging, again more strongly for minority students (*β* = 0.36, *p* < 0.001) compared to majority students (*β* = 0.17, *p* = 0.01). This means that all students—and especially minority students—reported higher school belonging if they also perceived high color‐evasion in school and a strong diversity climate in the classroom. As hypothesized, there was a significant interaction effect between color‐evasion and diversity climate for both, minority students (*β* = 0.14, *p* < 0.001) and majority students (*β* = 0.21, *p* < 0.001), meaning that the link between color‐evasion and school belonging was amplified in classrooms with a strong diversity climate (see Figures 2 and 3).

**Figure 4:**
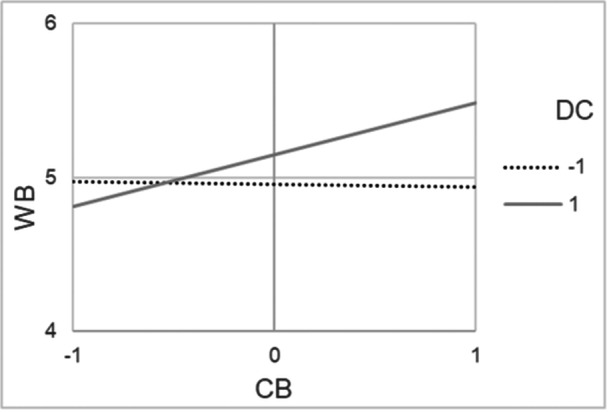
Interaction effects among cultural minority and majority students between perceived school‐level color‐evasion (CB) and diversity climate among classmates (DC) in the multigroup structural equation model with well‐being (WB) as outcome.

Regarding well‐being, the fully constrained model was accepted with a good fit (*χ* ^2^/df = 1.01, *p* = 0.43, RMSEA = 0.01 [90% CI from 0.00 to 0.10], CFI = 0.99), meaning that minority and majority students were comparable in their regression coefficients. Again, we found significant, positive direct effects only for color‐evasion and for both, minority students (*β* = 0.13, *p* = 0.03) and majority students (*β* = 0.13, *p* = 0.01). This means that all students reported higher general well‐being if they also perceived high color‐evasion in the classroom. Again, there was a significant interaction effect between color‐evasion and diversity climate for minority students (*β* = 0.13, *p* = 0.01) and majority students (*β* = 0.17, *p* = 0.01), meaning that also the link between color‐evasion and well‐being was amplified in classrooms with a strong diversity climate (see Figure 4).

Regarding intercultural contact, again the fully constrained model was accepted with a good fit (*χ* ^2^/df = 0.67, *p* = 0.72, RMSEA = 0.00 [90% CI from 0.00 to 0.08], CFI = 1.00), meaning that minority and majority students were comparable in their regression effects. Like school belonging, also for intercultural contact we found positive associations with color‐evasion and diversity climate, for minority students (color‐evasion: *β* = 0.22, *p* < 0.001; diversity climate: *β* = 0.33, *p* < 0.001) and majority students (color‐evasion: *β* = 0.26, *p* < 0.001; diversity climate: *β* = 0.42, *p* < 0.001). Therefore, students reported high intercultural contact if they also perceived high color‐evasion in the classroom and a strong diversity climate in school. No other relations or interactions included in the models were statistically significant (see Table 2).

### 3.3 Sensitivity Analyses

Due to the high ICC for intercultural contact (0.29), we performed additional multilevel analysis for this outcome variable, separating within‐person from classroom‐level effect while controlling for minority status. The final model had a perfect fit (*χ* ^2^/df = 0.00, *p* = 1.00, RMSEA = 0.00, CFI = 1.00). Results were largely comparable to multigroup SEM results, namely that students reported a higher intercultural contact if students individually perceived higher color‐evasion and diversity climate (color‐evasion: *β* = 0.17, *p* = 0.03; *β* = 0.24, *p* < 0.001), but also if they on average per classroom perceived higher color‐evasion and diversity climate (color‐evasion_class_: *β* = 0.88, *p* < 0.001; diversity climate_class_: *β* = 0.58, *p* < 0.001). This means that both individual students' and aggregated classroom perceptions of color‐evasion in the classroom and a diversity climate in school were positively related to intercultural contact. In addition, classroom‐level multiculturalism was negatively related to intercultural contact (*β* = −1.02, *p* < 0.001), meaning that students reported higher intercultural contact in their classroom if students within the same classroom on average perceived a lower multiculturalism.

## 4 Discussion

In this study, we investigated the relation between school diversity perspectives (multiculturalism, critical consciousness, color‐evasion) and student outcomes (school belonging, well‐being, intercultural contact; H1), we tested whether diversity climate among classmates could be a moderator (H2) and compared cultural minority and majority students (H3) while accounting for the nested structure of students in classrooms. Based on our findings, we draw several conclusions.

First, when looking at our three included school‐level diversity perspectives, only color‐evasion was robust in its positive relation to all three outcome variables for minority and majority students. Even though these results do not align with recent meta‐analytical findings (Bardach et al. 2024), they add to the canon of studies from Western Europe finding positive relations between color‐evasion and students' socioemotional and intergroup outcomes (e.g., Schwarzenthal et al. 2020). One reason could be that the Dutch national context has strongly shifted toward assimilative and restrictive migration policies (Jennissen et al. 2023; Meer et al. 2015; Scholten 2025). Here, color‐evasion may not only be more salient in educational discourse, but may also to some extent represent all students' wish for “blending in,” for being part of one coherent social group, and for not being called out as “the Other,” as may often be integral part of heritage culture learning in Dutch schools (Kennedy et al. 2023). With more nuanced measures, future studies may be better at adding gradation to and making a distinction between *equal treatment* and *ignoring difference* (e.g., Civitillo et al. 2021) and their meaning for marginalized and majority individuals embedded in an assimilative, restrictive educational and national climate as in the Netherlands.

This also means that the other two diversity perspectives (multiculturalism, critical consciousness) were unrelated to student outcomes, contrary to our predictions. One possible explanation is the arguably lower salience of these two perspectives in Dutch educational discourse in general, as mentioned above, and for cultural minority study participants in particular. Another possible explanation is that our measure of multiculturalism included two complementary, yet theoretically distinct forms of multiculturalism (Bardach et al. 2024), which may have canceled each other out in the model results: the form of *important differences* (e.g., Rosenthal and Levy 2010), in which the school wants students to better understand the lives, experiences, practices, and perspectives of diverse Others, and the form of *being affirmed in one's own culture* (e.g., Byrd 2017), in which schools want to pay attention to and integrate elements of all cultures represented in a classroom into everyday school practice. In our study, the majority of students reported a higher overlap between multiculturalism and critical consciousness, which may indicate that they interpreted multiculturalism items in the form of *important differences*. In contrast, minority students reported a higher overlap between multiculturalism and color‐evasion, which might indicate that they interpreted multiculturalism in the form of *being affirmed in one's own culture*, but specifically thinking about the Dutch majority culture (i.e., the shared culture among classmates) as one of their own cultures integrated in everyday school practice. This emphasizes the need to better account for bicultural or multicultural identifiers among minority students and to better explicate which cultures students have in mind when filling in survey items. Future studies will also have to elaborate on the coexistence of multiple diversity ideologies within schools, and especially on the distinction between the different forms of multiculturalism, even though these forms may not be easy to tease apart (see also Rosenthal and Levy 2010). This goal could be reached by employing multi‐informant and mixed‐methods study designs (e.g., Civitillo et al. 2017).

Second, diversity climate among classmates was robustly, directly, and positively related to two of the three outcomes (school belonging, intercultural contact) for minority and majority students. This aligns with earlier findings in professional workgroups (e.g., Hofhuis et al. 2012, 2016) and indicates that certain group‐level processes may be similar for professional and school contexts. Also, a strong diversity climate among classmates amplified (moderated) the link between color‐evasion with socioemotional outcomes (school belonging, well‐being; i.e., “feeling well”). Thus, in classrooms, fostering an environment of openness for and appreciation of diversity—among students but also from teacher to student—may ensure that school‐level ideologies on how to best manage diversity (i.e., school diversity perspectives) translate into favorable and comparable school experiences for cultural minority and majority students in schools. Importantly, this highlights the added value of disentangling school‐level and classroom‐level indicators of approaching diversity, and their unique contributions to student experiences and outcomes.

Lastly, school diversity perspectives and diversity climate among classmates were more strongly related with school belonging for cultural minority compared to majority students. Yet, both predictors were also unrelated to one another for minority students. First, this may indicate that school‐level and classroom‐level approaches to diversity may uniquely and individually contribute especially to minority students' social position in school, given that minority students are likely to feel disrespected, less attached, or more alienated from school compared to their majority counterparts due to societal pressures and stereotypes (Steele 1997). Second, this may indicate that cultural minority students perceive that school‐level diversity perspectives operate independently from the quality of interactions among classmates (i.e., openness and appreciation of cultural diversity). In other words, what a school wants may not directly translate into what minority students see in their classmates' behaviors. Given the hypothesized central function of school belonging for minority students' academic development (Murphy and Zirkel 2015), it is therefore crucial to simultaneously intervene on the school level and classroom level to ensure the optimal conditions for all students' school belonging and well‐being.

### 4.1 Limitations and Future Research

Despite the contributions of this study to the field of educational psychology, we must mention some limitations. First, our study did not take into account the cultural composition of schools and classrooms, which may be a relevant factor related to intergroup outcomes. For example, color‐evasion/colorblindness is likely related to more negative intergroup outcomes in studies with larger proportions of majority students (Bardach et al. 2024). Second, future research may also include the rather understudied *polyculturalism perspective* which emphasizes the connections among groups due to historical and present interactions (e.g., Rosenthal and Levy 2010) and may uniquely contribute to students' socioemotional development in school (Juang et al. 2020). Third, relying solely on student reports, we were unable to examine school‐level mechanisms more holistically, for example, by adding teacher‐level variables (e.g., teacher diversity perspectives, culturally sensitive teaching practices). Teachers are important facilitators of an open and appreciative diversity climate in the classroom (Gay 2018), translating school‐level visions into visible classroom‐level behaviors while responding to students' needs to be seen and heard. Importantly, perceived teacher support is a strong predictor of school belonging (Allen et al. 2018). Future studies are therefore advised to expand their scope, to move beyond student reports, and toward multi‐informant and mixed‐methods designs (e.g., Civitillo et al. 2017). Finally, the small sample size and cross‐sectional nature of this study did not allow for simultaneously testing multigroup and multilevel effects and for testing causality, which are clear limitations. Still, the findings of our study provide robust indicators that school‐level diversity perspectives and diversity climate among classmates may have divergent effects and may even interact with each other to predict student outcomes, especially when considering students identifying with minoritized societal groups. We hope that our work may inspire further large‐scale and longitudinal studies which triangulate the perspectives from different agents (teachers, students), which may uncover these effects in greater detail.

## 5 Conclusion

This study uniquely combined organizational psychological approaches with educational approaches to cultural diversity perspectives and cultural diversity climate to measure what can contribute to more equitable student experiences in secondary schools. Our results stress the importance of disentangling effects, but also of sharpening our assumptions and measures regarding school‐level effects (e.g., diversity perspectives) and classroom‐level effects (e.g., diversity climate) for explaining inequities in socioemotional outcomes (“feeling well”) and intergroup outcomes (“doing well”; Suárez‐Orozco et al. 2018) between cultural and ethnic minority and majority students in Western Europe.

## Ethics Statement

This work was approved by the Ethical Committee of the Erasmus University Rotterdam (Application Number ETH2223‐0486).

## Conflicts of Interest

The authors declare no conflicts of interest.

## Peer Review

The peer review history for this article is available at https://www.webofscience.com/api/gateway/wos/peer-review/10.1002/jcop.70082.

## Data Availability

The authors have nothing to report.

## Endnotes

1 Our distinction between diversity climate and diversity perspectives is rooted in the theoretical and empirical tradition of organizational research (Hofhuis and Vietze 2025; Leslie and Flynn 2022). We acknowledge that this is different from a some recent studies in educational research, which conceptualize cultural diversity climate as approaches for addressing cultural diversity in school settings (e.g., multiculturalism, colorblindness; Bardach et al. 2024) and how these approaches manifest in the practices, policies, norms, and general atmosphere in classrooms and schools, shape interactions among students as well as between students and teachers or students and other school personnel (e.g., Schachner et al. 2021).
